# Aberrantly expressed Wnt5a in nurse-like cells drives resistance to Venetoclax in chronic lymphocytic leukemia

**DOI:** 10.1038/s41420-022-00884-y

**Published:** 2022-02-24

**Authors:** Yao Guo, Hanzhong Pei, Bo Lu, Dengyang Zhang, Yuming Zhao, Fuqun Wu, Honghua Sun, Junbin Huang, Peng Li, Chenju Yi, Chengming Zhu, Yihang Pan, Shunjie Wu, Chun Chen, Xiaojun Xu, Yun Chen

**Affiliations:** 1 Edmond H. Fischer Translational Medical Research Laboratory, Scientific Research Center, The Seventh Affiliated Hospital, Sun Yat-sen University, Shenzhen, 518107 Guangdong China; 2 Department of Hematology, The Seventh Affiliated Hospital, Sun Yat-sen University, Shenzhen, 518107 Guangdong China; 3 Clinical laboratory, The Seventh Affiliated Hospital of Sun Yat-sen University, Shenzhen, 518107 Guangdong China; 4 Department of Pediatrics, The Seventh Affiliated Hospital, Sun Yat-sen University, Shenzhen, 518107 Guangdong China

**Keywords:** Chronic lymphocytic leukaemia, Cancer microenvironment

## Abstract

Chronic lymphocytic leukemia (CLL) is characterized by the accumulation of neoplastic B lymphocytes with high levels of Wnt5a in the plasma. Currently, the cell source of Wnt5a remains controversial. The receptor of Wnt5a is ROR1, whose expression is associated with disease progression and resistance to venetoclax, a BCL-2 inhibitor approved for the treatment of CLL. In this study, we found that the levels of Wnt5a in the plasma of CLL patients were positively correlated with absolute monocyte counts, but not lymphocyte counts. We cultured monocyte-derived nurse-like cells (NLCs) from patients with CLL, and detected Wnt5a expressed in NLCs. Flow cytometry and transwell assays showed that the antibody neutralizing Wnt5a inhibited the enhanced survival and migration in CLL cells co-cultured with NLCs. Furthermore, we performed a drug screening with CLL cells cultured with or without NLCs with a library containing 133 FDA-approved oncology drugs by using high-throughput flow cytometry. We observed a significant resistance to venetoclax in CLL cells co-cultured with NLCs. Immunoblot revealed the activation of NF-κB with enhanced expression of MCL-1 and BCL-XL in CLL cells co-cultured with NLCs. Neutralizing Wnt5a or blocking NF-κB pathway significantly decreased the expression of MCL-1 and BCL-XL, which leads to enhanced sensitivity to venetoclax in CLL cells co-cultured with NLCs. In conclusion, our data showed that NLCs could be one of the sources of Wnt5a detected in patients with CLL, and Wnt5a-induced NF-κB activation in the CLL microenvironment results in resistance to venetoclax in CLL cells.

## Introduction

Chronic lymphocytic leukemia (CLL) is a malignancy of B lymphocytes that is characterized by the accumulation of small, mature-appearing neoplastic lymphocytes with CD5 expression in the blood, bone marrow, and secondary lymphoid tissues [1]. It is the most common leukemia in the Western world with a median age of diagnosis around 70. In the United States, there are approximately 20,000 new cases and 4000 deaths from CLL per year [2]. Despite the significant progress in chemo-immunotherapy and inhibitors targeting Bruton’s tyrosine kinase (BTK) made over the past decade [3–5], some patients with high-risk features still experience refractory or progressive disease due to the development of drug resistance. Novel strategies overcoming resistant mechanisms are urgently needed.

Venetoclax is a potent small molecule inhibitor targeting BCL-2, an anti-apoptotic protein highly expressed in CLL cells [6]. Treatment with venetoclax has been shown to be effective with limited toxicity in patients with CLL with 72% overall response and 19.4% complete remission in real-world setting [7]. However, although remissions are durable, patients treated with venetoclax always experience relapses eventually. Previous studies found that venetoclax is highly potent against unstimulated CLL cells in vitro. However, leukemic cells activated with signals mimicking microenvironment stimuli are less sensitive to the drug [8, 9]. These studies indicate a critical role of CLL microenvironment in the resistance to venetoclax, but the detailed mechanisms are not fully uncovered.

In the bone marrow and secondary lymphoid tissues, CLL cells engage in complex cellular and molecular interactions with non-neoplastic accessory cells in the microenvironment [10–12]. These interactions provide CLL cells survival, proliferation, and drug resistance. Nurse-like cells (NLCs) are key components in the microenvironment that have well-documented protective effects to CLL cells [13–15]. They have been shown to attract CLL cells by the release of chemokines, such as CXCL12 and CXCL13 [16]. They further secrete proteins, such as B-cell activating factor (BAFF) and a proliferation-inducing ligand (APRIL), which enhance CLL cell survival [17]. However, inhibiting these factors cannot abolish the supportive effects of NLCs completely [18], suggesting the involvement of other factors.

Receptor tyrosine kinase-like orphan receptor 1 (ROR1) is an oncoembryonic antigen expressed on CLL cells, but not on most normal postpartum tissues [19]. The ligand that binds to ROR1 is Wnt5a, which we find present at high levels in the plasma of patients with CLL relative to that of age-matched healthy adults [20, 21]. Wnt5a can induce ROR1-dependent cell signals, thereby enhancing leukemia-cell proliferation, migration, and survival [21–27]. Previous studies showed autocrine of Wnt5a in neoplastic B cells [28], whereas *WNT5A* transcripts only present in neoplastic B cells in around 38% of patients with CLL [29], and high-levels of Wnt5a could be detected universally in their plasma [20, 21], suggesting cell-sources other than CLL neoplastic cells. We previously found that NLCs could release this non-canonical Wnt factor [13], but whether NLCs serve as the main source of Wnt5a in plasma of patients with CLL remains unknown, and the function of NLCs-released Wnt5a in the mechanisms of drug resistance still needs to be investigated. In the present study, we examined the expression of Wnt5a in NLCs in comparison with neoplastic CLL cells in patients and explored the involvement of Wnt5a in NLCs-induced survival, migration, and venetoclax-resistance in CLL cells.

## Results

### Monocyte-derived NLCs correlate with Wnt5a levels in plasma from CLL patients

**Fig. 1:**
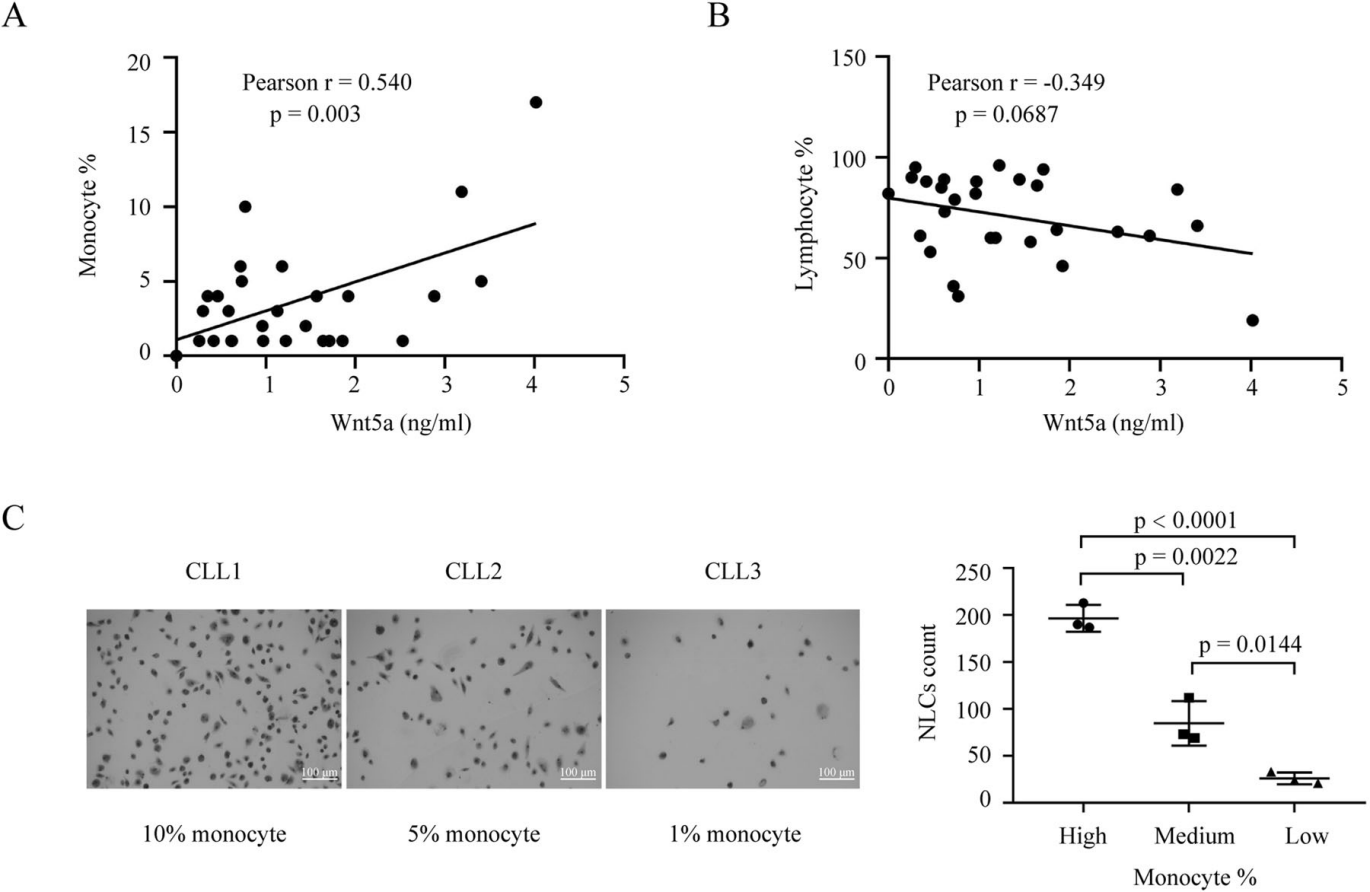
Monocyte-derived NLCs correlate with Wnt5a levels in plasma from CLL patients. **A**, **B** The concentration of Wnt5a in plasma of patients positively correlates with their monocyte percentage in whole white blood cells (**A** *n* = 28, Pearson correlation = 0.540, *p* = 0.003), but not their lymphocyte percentage (**B** *n* = 28, Pearson correlation = −0.349, *p* = 0.0687). **C** Wright–Giemsa staining of NLCs cultured from PBMCs of patients with CLL with different monocyte percentage. Dot figures represent numbers of NLCs in the field (40X) of each group. Error bars denote standard deviation (*n* = 3).

NLCs are derived from circulating monocytes from blood of patients with CLL [15]. We analyzed whole blood counts and levels of Wnt5a in plasmas of 28 CLL patients by ELISA. The result showed that levels of Wnt5a had a positive correlation with monocyte percentage, but not lymphocyte percentage (Fig. 1A, B), suggesting that Wnt5a was from monocytes or monocyte-derived cells instead of neoplastic B cells in patients with CLL. We cultured NLCs using PBMCs from patients with different monocyte counts and found that PBMCs with high monocyte counts generated high number of NLCs (Fig. 1C). We hypothesized that high monocyte counts could result in high number of NLCs, which eventually led to high levels of Wnt5a in plasma of patients with CLL. These data indicate that monocyte-derived NLCs could be the main source of high levels of Wnt5a in patients with CLL.

### NLCs express high-levels of Wnt5a compared with neoplastic leukemia cells

**Fig. 2:**
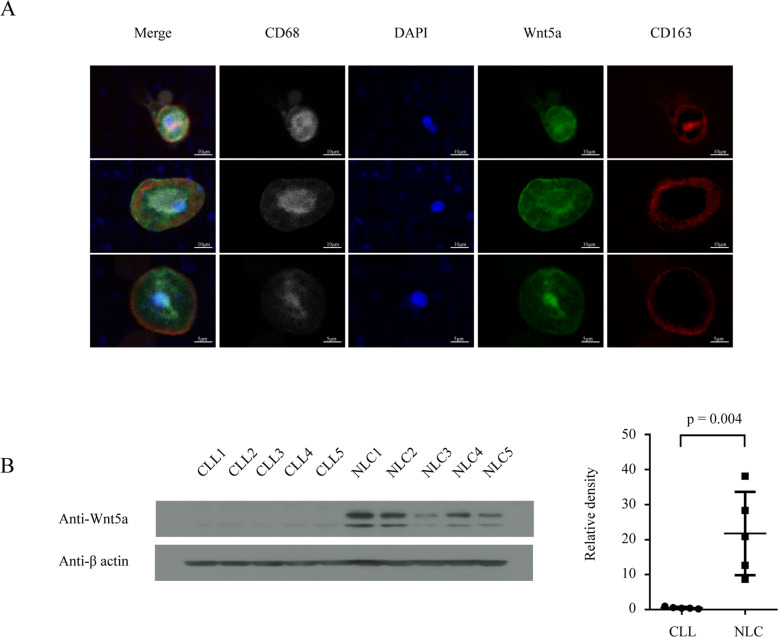
NLCs but not neoplastic leukemia cells express Wnt5a. **A** Fluorescent microscopy of Wnt5a expressed by NLCs. CD163 and CD68 were used as markers for NLCs. **B** Immunoblot analysis of Wnt5a in paired NLCs and CLL cells. Dot figures represent ratios of densities (Wnt5a/β-actin). Error bars denote standard deviation (*n* = 5).

For this study, we generated NLCs from blood mononuclear cells of CLL patients using established methods [14]. NLCs were isolated free of leukemia cells via adherence. Ultra-high resolution confocal fluorescent microscopy of NLCs showed that Wnt5a was expressed in NLCs (Fig. 2A). In addition, we measured Wnt5a levels in cell lysates from purified CLL cells or NLCs of the same patient with immunoblot. The result showed that Wnt5a was highly expressed in NLCs but neglectable in CLL cells (Fig. 2B).

### Wnt5a is involved in NLCs-induced survival and migration of CLL cells

**Fig. 3:**
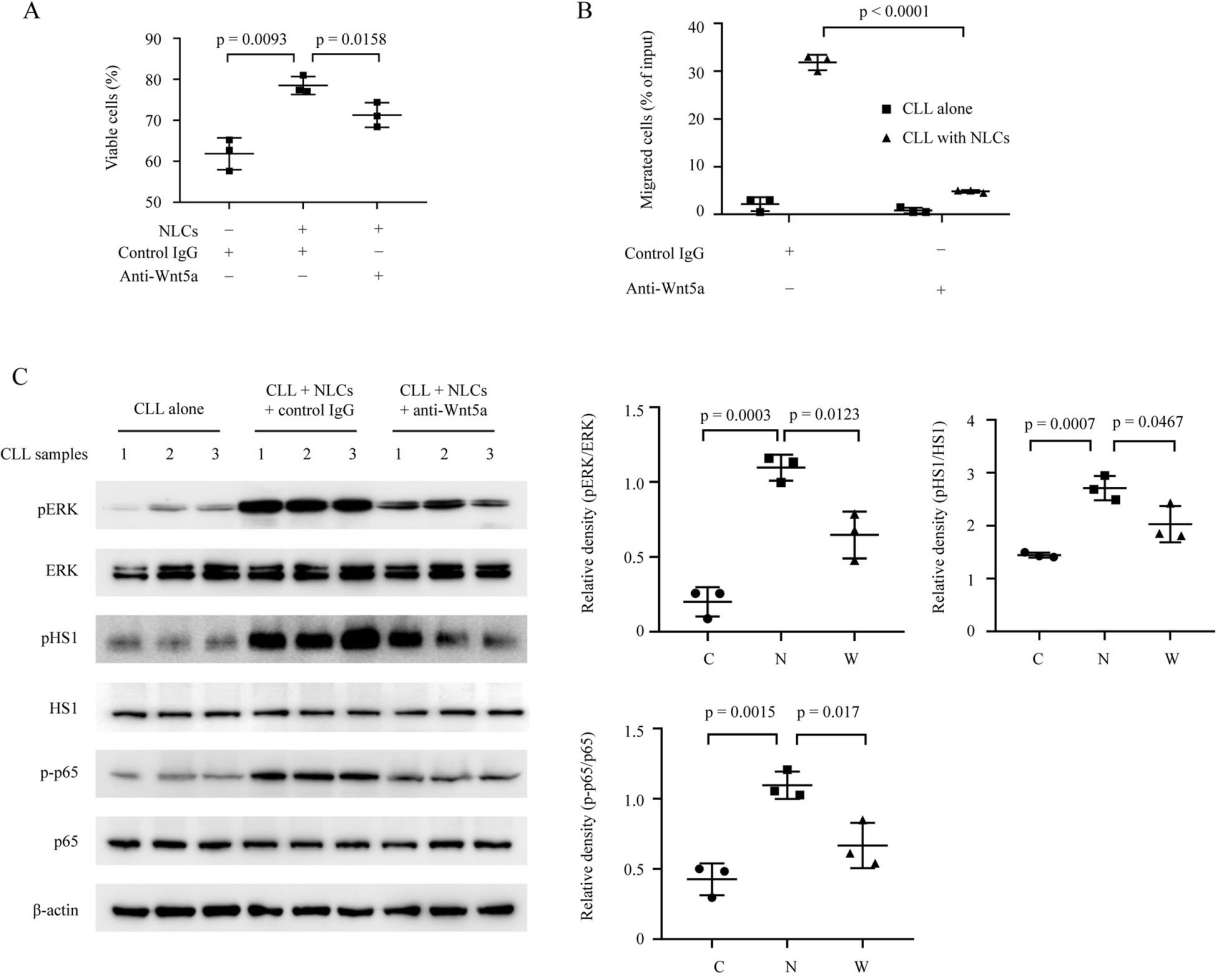
Wnt5a is involved in NLCs-induced survival and migration of CLL cells. **A** Cell viability analysis by trypan blue staining of CLL cells with or without NLCs treated by anti-Wnt5a or control mAb of irrelevant specificity for 24 h (*n* = 3). **B** Percentage of CLL cells migrating toward NLCs. CLL cells were treated by anti-Wnt5a or control mAb of irrelevant specificity for 4 h. Error bars denote standard deviation (*n* = 3). **C** Immunoblot analysis of phospho-ERK, total ERK, phospho-HS1, total HS1, phospho-p65, and total p65 in lysates of CLL cells with or without NLCs treated by anti-Wnt5a or control mAb of irrelevant specificity for 24 h. Dot figures represent ratios of densities (pERK/ERK, pHS1/HS1, and p-p65/p65).

NLCs can attract CLL cells and protect them from apoptosis by elaborating factors including CXCL12, CXCL13, BAFF, and APRIL [1]. We hypothesized that Wnt5a expressed by NLCs could also be involved in these effects. We used trypan blue staining to assess the viability of CLL cells cultured with or without NLCs, and with or without the antibody neutralizing Wnt5a. We found that anti-Wnt5a, but not a mAb of irrelevant specificity, could significantly inhibit anti-apoptotic effects of CLL cells when co-cultured with NLCs (Fig. 3A). Next, we performed trans-well assays to assess migration of CLL cells when cultured with or without NLCs, and with or without a neutralizing mAb specific for Wnt5a. As the result, anti-Wnt5a could significantly inhibit migration of CLL cells enhanced by co-culture with NLCs (Fig. 3B). We also examined phosphorylation of ERK, HS1, and p65 in CLL cells co-cultured with NLCs, which account for Wnt5a-induced survival and migration of CLL cells according to our previous studies [13, 23, 30]. We found that CLL cells co-cultured with NLCs, but not CLL cells cultured alone, had high-level of phosphorylated ERK, HS1, and p65. The phosphorylation of ERK, HS1, and p65 in co-cultured CLL cells and cell migration could be blocked by neutralizing anti-Wnt5a, but not by control mAb of irrelevant specificity (Fig. 3C).

### NLCs lead to drug resistance of CLL cells in a high-throughput screening

**Fig. 4:**
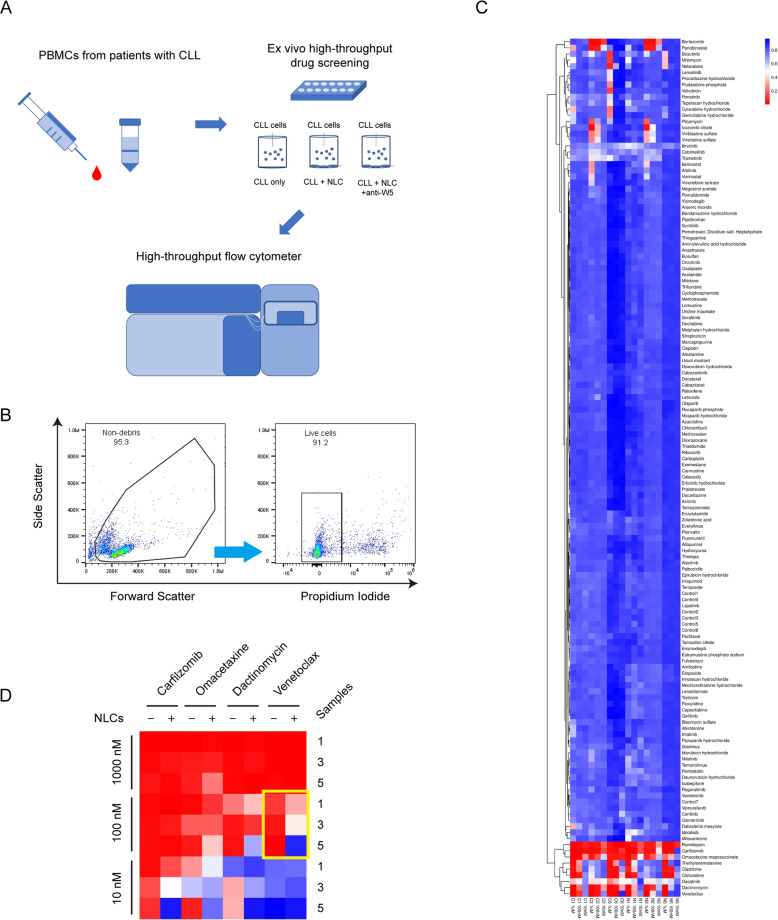
Wnt5a is involved in NLCs-induced drug-resistance of CLL cells. **A** The flow chart of the high-throughput drug sensitivity analysis. **B** The gating strategy of live cells in flow cytometry. **C** The heatmap of the high-throughput drug sensitivity analysis by using 133 FDA-approved oncology drugs. C1, 2, and 5: CLL cells cultured alone as the control group. N1, 2, and 5: CLL cells cultured with NLCs. **D** The sensitivity of CLL cells from patients 1, 2, and 5 to dactinomycin, carfilzomib, omacetaxine mepesuccinate, and venetoclax. The yellow box highlights the resistance to venetoclax of CLL cells co-cultured with NLCs.

We used high-throughput flow cytometry to evaluate the drug-sensitivity of primary CLL cells cultured with or without NLCs in three patients with CLL (Fig. 4A, B). A library composed of 133 FDA-approved oncology drugs was used for the high-throughput screening. We found that romidepsin, carfilzomib, omacetaxine mepesuccinate, dasatinib, venetoclax, and dactinomycin potently inhibited CLL cells (Fig. 4C). Co-culture of CLL cells with NLCs decreased the potency of these drugs, especially carfilzomib, omacetaxine mepesuccinate, dactinomycin, and venetoclax (Fig. 4D). Note that only venetoclax among these drugs is approved for the treatment of CLL by FDA.

### Wnt5a upregulated MCL1 and BCL-XL in CLL cells co-cultured with NLCs

**Fig. 5:**
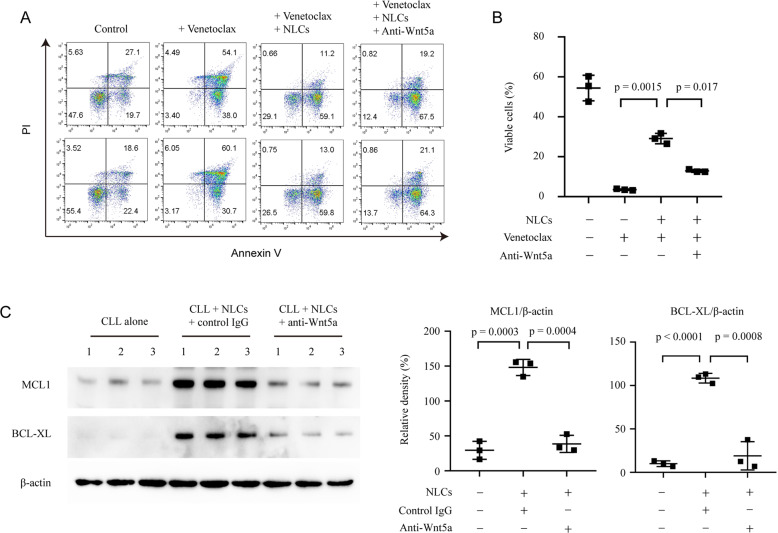
NLCs upregulate MCL1 and BCL-XL in CLL cells through Wnt5a. **A** Flow cytometry analysis of apoptosis of CLL cells cultured with venetoclax, NLCs, and anti-Wnt5a for 24 h. Dot figures represent viable cells in each group (*n* = 3). **B** Immunoblot analysis of MCL1 and BCL-XL in lysates of CLL cells with or without NLCs treated by anti-Wnt5a or control mAb of irrelevant specificity for 24 h. Dot figures represent ratios of densities (MCL1/β-actin and BCL-XL/β-actin).

We used flow cytometry to analyze apoptosis of CLL cells induced by venetoclax with or without NLCs and mAb neutralizing Wnt5a. We found that NLCs significantly protected CLL cells from apoptosis, which is consistent with our high-throughput drug sensitivity screening (Fig. 5A, B). Furthermore, mAb neutralizing Wnt5a inhibited the protective effect of NLCs in CLL cells treated by venetoclax (Fig. 5A, B). Immunoblot showing that CLL cells co-cultured with NLCs have significant upregulation of MCL1 and BCL-XL (Fig. 5C, D), which are BCL2 family members involved in drug resistance of venetoclax. These effects were blocked by mAb neutralizing Wnt5a, indicating an important role of Wnt5a in NLCs-induced protective effects against venetoclax in CLL cells.

### Wnt5a induced resistance to venetoclax through activation of NF-κB in CLL cells

**Fig. 6:**
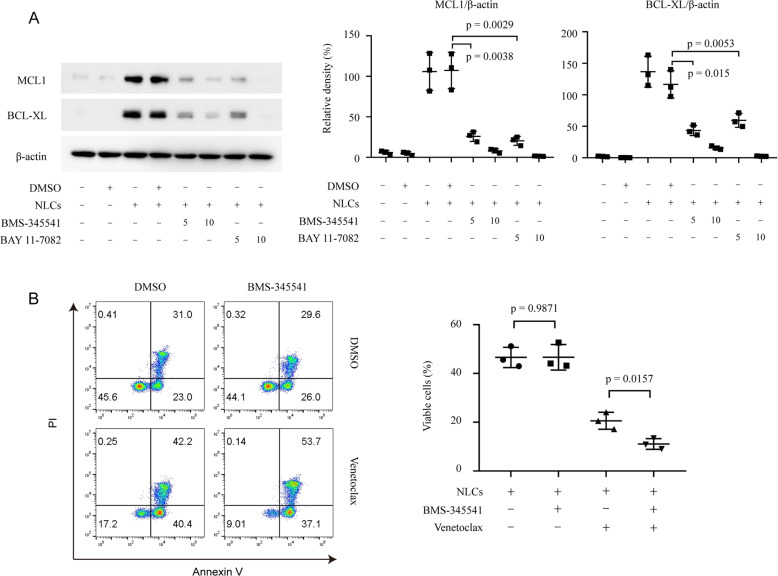
NF-κB activation is required in NLCs-induced resistance to venetoclax in CLL cells. **A** Immunoblot analysis of MCL1 and BCL-XL in lysates of CLL cells with or without NLCs treated by BMS-345541 or BAY 11–7082. Bar figures represent ratios of densities (MCL1/β-actin and BCL-XL/β-actin, *n* = 3). **B** Flow cytometry analysis of apoptosis of CLL cells cultured with NLCs with 50 nM venetoclax and 5 μM BMS-345541. Dot figures represent the percentage of viable cells in each group (*n* = 3).

We previously reported that Wnt5a could activate NF-κB pathway in CLL cells [13], which might serve as a mechanism to upregulate MCL1 and BCL-XL [31, 32]. We found that inhibition of NF-κB pathway by small molecule inhibitors significantly downregulated the expression of MCL1 and BCL-XL in CLL cells co-cultured with NLCs by immunoblot (Fig. 6A). In addition, treatment with NF-κB inhibitor significantly blocked the protective effect of NLCs against venetoclax in CLL cells by flow cytometry (Fig. 6B). Note that NF-κB inhibitor BMS-345541 alone did not change the cell viability of CLL cells.

## Discussion

**Fig. 7:**
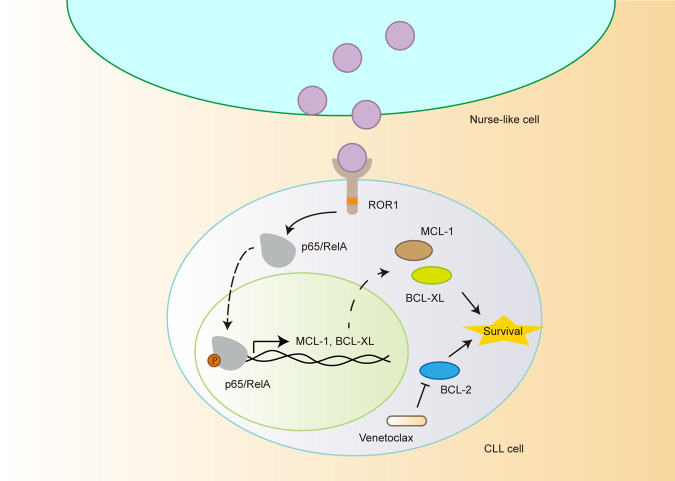
The model for resistance to venetoclax in CLL cells. NLCs-Wnt5a-induced activation of NF-κB drives resistance to venetoclax in CLL cells through upregulation of MCL1 and BCL-XL.

In the present study, we found that aberrantly expressed Wnt5a in NLCs leads to resistance to venetoclax in CLL through upregulation of MCL1 and BCL-XL via NF-κB activation. First, we found that aberrantly high levels of Wnt5a in CLL patients were correlated with monocyte counts, but not lymphocyte counts, suggesting monocyte-derived cells as main sources of Wnt5a. In addition, we identified that monocyte-derived NLCs express Wnt5a. Neutralizing Wnt5a significantly inhibited NLCs-induced survival, migration, and corresponding cell signals in CLL cells. Furthermore, with a high-throughput drug screening assay, we identified significant drug resistance to venetoclax in CLL cells co-cultured with NLCs. Finally, we found that Wnt5a-induced upregulation of MCL1 and BCl-XL by activation of NF-κB is involved in resistance to venetoclax in CLL cells co-cultured with NLCs (Fig. 7).

Wnt5a activates non-canonical WNT signaling pathways, through binding to different members of the Frizzled- and ROR-family receptors [33]. Studies have shown that Wnt5a signaling is emerging as an important event in malignancies, including cancer cell proliferation, invasion, metastasis, metabolism, inflammation, and chemo-resistance [34]. Also, Wnt5a signaling is reported to modulate the interaction between cancer cells and accessory cells in the microenvironment, contributing to disease progression in solid tumors. In gastric carcinoma, microenvironmental lymphocytes secrete Wnt5a to stimulate the proliferation of malignant cells by activating RhoA [35]. In colon cancer, enhanced stromal Wnt5a expression promotes directional migration and invasion of cancer cells [36]. In this study, we identified that Wnt5a was involved in the crosstalk between NLCs and CLL cells. Blocking Wnt5a signaling inhibited NLCs-induced CLL proliferation, migration, and drug resistance, suggesting Wnt5a signaling as a potential therapeutic target in the microenvironment of CLL cells.

The emergence of targeted therapy for patients with CLL has altered the therapeutic landscape of the disease, but the drug resistance remains a challenge for recurrent and relapsed CLL [5, 37, 38]. Our ex vivo high-throughput drug screening found a drug-resistance to venetoclax in CLL cells induced by NLCs. Venetoclax is a selective BCL-2 inhibitor that has been approved for the treatment of first-line and relapsed/refractory CLL [6]. Monotherapy with venetoclax facilitates a rapid reduction in the disease burden with a high overall response of about 80% and a complete response of 6–20% in patients with relapsed or refractory CLL, including those with poor prognosis [39]. However, the resistance to venetoclax can be found in some CLL patients with mechanisms not fully uncovered [38]. Our study observed NLCs-induced CLL resistance to venetoclax, which was inhibited by anti-Wnt5a antibody. These data provide novel mechanisms for venetoclax resistance and potential therapeutic options overcoming the resistance.

Previously, we reported that Wnt5a presents at high levels in patients with CLL compared with health donors [21, 40]. We speculate that the high-level Wnt5a noted in the plasma of patients with CLL is produced primarily by NLCs, which reside in lymphoid tissues. If so, then Wnt5a present in the plasma of patients with CLL may extend the protective effects of NLCs beyond the CLL microenvironment in which NLCs reside. Treatment targeting Wnt5a-signaling may mitigate such effects, potentially enhancing the clearance of leukemia cells when used alone or in combination with drugs that target other survival-signaling pathways in CLL.

## Methods

### CLL specimens

Blood samples were collected from The Seventh Affiliated Hospital, Sun Yat-sen University. Patients were provided written informed consent using a protocol approved by the Institutional Review Board of The Seventh Affiliated Hospital, Sun Yat-sen University, in accordance with the Declaration of Helsinki. Peripheral blood mononuclear cells (PBMCs) were isolated by density-gradient centrifugation with Ficoll-Paque PLUS (GE Healthcare Life Sciences, PA, USA), followed by purification with human B Cell Isolation Kit (130-091-151, Miltenyi Biotec Inc., CA, USA). Plasma was collected from blood samples that had undergone centrifugation for 10 min at 187 × *g* and stored at −80 °C.

### Generation of nurse-like cells

PBMCs isolated by Ficoll-Paque PLUS from CLL patients were suspended in RPMI with 20% FBS to a final concentration of 2 × 10^7^/ml, as described [13]. After 14 days, the non-adherent CLL cells were removed by vigorously pipetting the contents of the well, leaving the adherent cells untouched. We observed these cells to have the morphology typical of NLC via microscopy.

### Materials

133 FDA-approved oncology drugs were selected from FDA-approved Drug Library (L1300) of Selleckchem (TX, USA).

### Immunoblot analysis

Immunoblot analysis was performed as described [41]. Primary mAb for β-actin (#4970), ERK (#4695), phospho-ERK (Thr202/Tyr204) (#4370), HS1 (#3892), phospho-HS1 (Tyr397) (#8714), p65 (#8242), phospho-p65 (Ser536) (#3033), MCL-1 (#5453), and BCL-XL (#15071) were from Cell Signaling Technology (MA, USA). mAb (MAB645) for Wnt5a was from R&D Systems (MN, USA). Secondary antibody conjugated with horseradish peroxidase was obtained from Cell Signaling Technology. The original western blots are provided (Supplemental Material, Supplementary Full Blots).

### Apoptotic analysis

Apoptotic analysis was performed as described [42]. CLL cells were cultured with or without NLCs or anti-Wnt5a antibody (MAB645; R&D Systems) (5 μg/mL) in RPMI culture-media, which were analyzed by Annexin V FITC Apoptosis Detection Kit I (BD Biosciences) and FACSCalibur Flow Cytometer (BD Biosciences). FlowJo (V10, FlowJo, OR, USA) was used for the analysis of apoptosis.

### Fluorescent microscopy

Cells were stained with anti-CD163-PE (BD Biosciences, CA, USA) at 4 °C for 20 min, washed twice with FACS buffer, and then fixed with 4% paraformaldehyde for 20 min at 4 °C. Cells were washed twice with PBS and permeabilized with 0.3% Triton X-100 in PBS at 4 °C for 10 min. After the cells were washed twice with PBS, they were blocked with 5% BSA in PBS at 4 °C for 30 min. Anti-Wnt5a-FITC (Aviva Systems Biology, CA, USA) and anti-CD68-perCP (BioLegend, CA, USA) were added in blocking buffer and incubated overnight at 4 °C. Cells were washed twice with PBS and incubated at room temperature for 60 min. Cells were washed twice with PBS, and DAPI was added and incubated at room temperature for 10 min. Images were collected on a Nikon A1R confocal microscope.

### Migration assay

The migration assay across 5.0 μm pore size polycarbonate membranes was performed as described [21]. A total of 5 × 10^5^ cells were starved overnight and seeded in the upper compartment of transwell inserts (Corning, NY, USA). Cells were incubated for 4 h in serum-free medium with mAb neutralizing Wnt5a or control IgG at 37 °C and 5% CO_2_, and the migration toward NLCs was analyzed by microscopy. The percentage of migrating cells was calculated as the number of migrated cells in response to chemokine divided by the total number of input cells.

### ELISA

Wnt5a ELISA kit (Aviva Systems Biology, CA, USA) was used to measure Wnt5a levels in plasma from patients with CLL per the manufacturer’s instruction.

### High-throughput drug sensitivity analysis

Primary CLL cells were cultured alone or with NLCs with RPMI with 10% FBS in 96-well plates. 133 oncology drugs were added into wells with final concentrations of 10 nM, 100 nM, and 1000 nM. CLL cells cultured with drugs or DMSO (<0.1%) as the vehicle were maintained in a humidified atmosphere at 37 °C with 5% CO_2_. Cells were stained with 0.5 μg/ml Propidium Iodide before subjected to analysis. The Stratedigm S1400Exi flow cytometer platform with A600 plate loader was used for high-throughput flow cytometry analysis. FlowJo (V10, FlowJo, OR, USA) was used for the analysis of percentage of live cells and R version 3.5.3 with pheatmap package (version 1.0.12) was used to generate heatmaps.

### Statistical analyses

Data are presented as the mean ± SD. Differences between two groups were determined by two-tailed Student’s t-test. Correlation between two groups was determined by Pearson correlation coefficients. Significance was analyzed by GraphPad Prism 6.0 (GraphPad Software Inc., CA, USA) and *p* values of less than 0.05 were considered significant.

## Supplementary information

Supplemental Material

## Data Availability

All data generated or analyzed during this study are included in this published article and its supplementary information files.
