# Supplementary material for: Aberrantly expressed Wnt5a in nurse-like cells drives resistance to Venetoclax in chronic lymphocytic leukemia
Source: Cell Death Discov. 2022 Feb 24;8:82. doi: 10.1038/s41420-022-00884-y (PMC8873424; doi:10.1038/s41420-022-00884-y)

**SUPPLEMENTAL INFORMATION**

**Aberrantly Expressed Wnt5a in Nurse-Like Cells Drives Resistance to Venetoclax in Chronic Lymphocytic Leukemia**

Yao Guo^1*^, Hanzhong Pei^1*^, Bo Lu^2^*, Dengyang Zhang^1^, Yuming Zhao^1^, Fuqun Wu^3^, Honghua Sun^3^, Junbin Huang^4^, Peng Li^1^, Chenju Yi^1^, Chengming Zhu^1^, Yihang Pan^1^, Shunjie Wu^2^, Chun Chen^4#^, Xiaojun Xu^2#^, Yun Chen^1#^

^1^Edmond H. Fischer Translational Medical Research Laboratory, Scientific Research Center, The Seventh Affiliated Hospital, Sun Yat-sen University, Shenzhen, 518107 Guangdong, China.

^2^Department of Hematology, The Seventh Affiliated Hospital, Sun Yat-sen University, Shenzhen, 518107, Guangdong, China.

^3^Clinical laboratory, The Seventh Affiliated Hospital of Sun Yat-sen University, Shenzhen, 518107 Guangdong, China.

^4^Department of Pediatrics, The Seventh Affiliated Hospital, Sun Yat-sen University, Shenzhen, 518107, Guangdong, China.

^#^Correspondence: Dr. Yun Chen, Edmond H. Fischer Translational Medical Research Laboratory, Scientific Research Center, The Seventh Affiliated Hospital, Sun Yat-sen University, Shenzhen, 518107 Guangdong, China; phone: (0755)81207021; email: cheny653@mail.sysu.edu.cn; ORCID: 0000-0002-8348-8755

Dr. Xiaojun Xu, Department of Hematology, The Seventh Affiliated Hospital, Sun Yat-sen University, Shenzhen, 518107, Guangdong, China. phone: (0755)81206772; email: xuxj29@mail.sysu.edu.cn; ORCID: 0000-0001-5030-7201

Dr. Chun Chen, Department of Pediatrics, The Seventh Affiliated Hospital, Sun Yat-sen University, Shenzhen, 518107, Guangdong, China. phone: (0755)81206752; email: chenchun@mail.sysu.edu.cn; ORCID: 0000-0002-9800-4012

^*^These authors contributed equally to this work.

**Running title:** NLCs lead to venetoclax resistance in CLL

**Supplementary Full Blots**

Whole blots in Figure 2


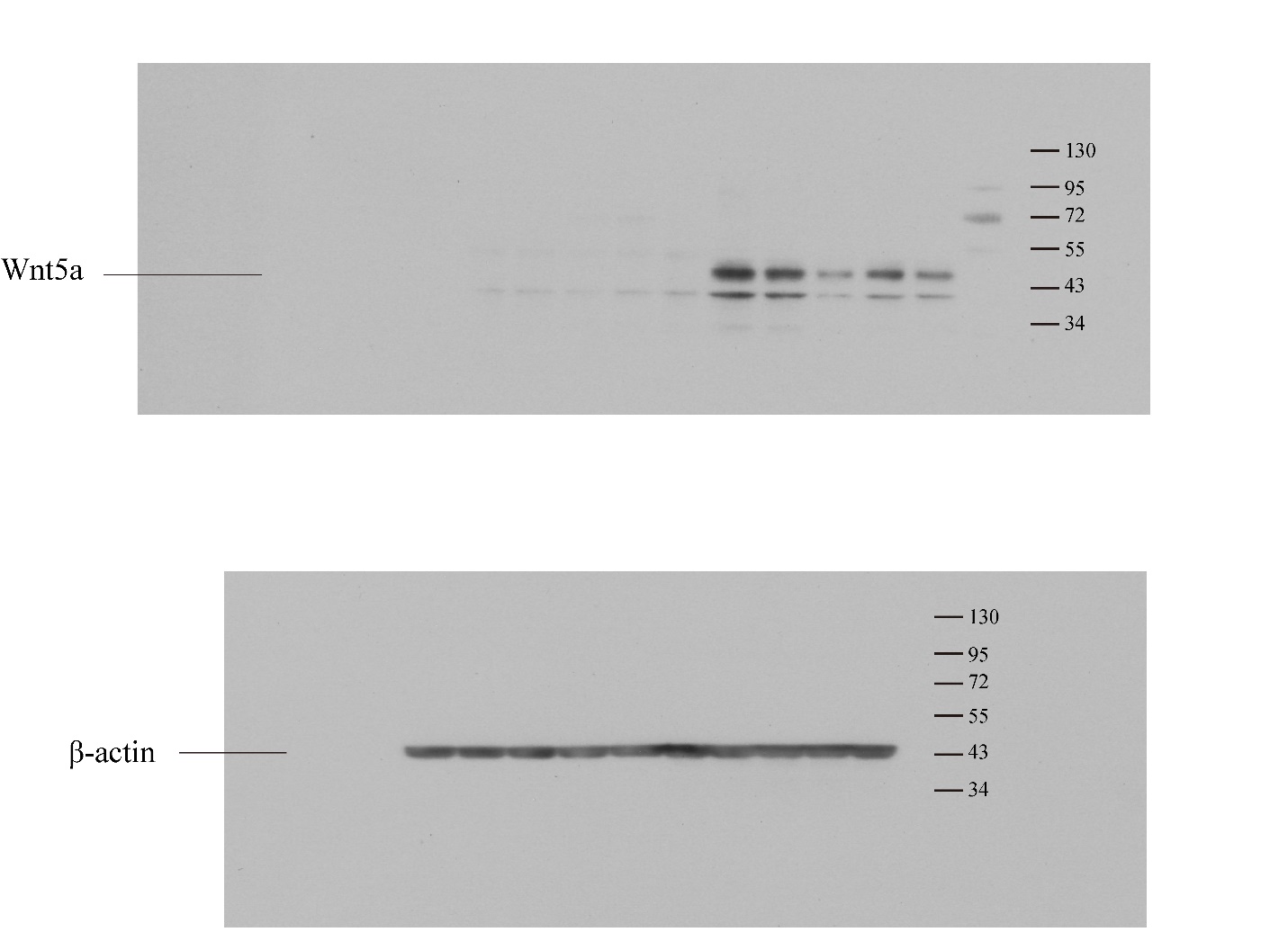


Full blots in Figure 3


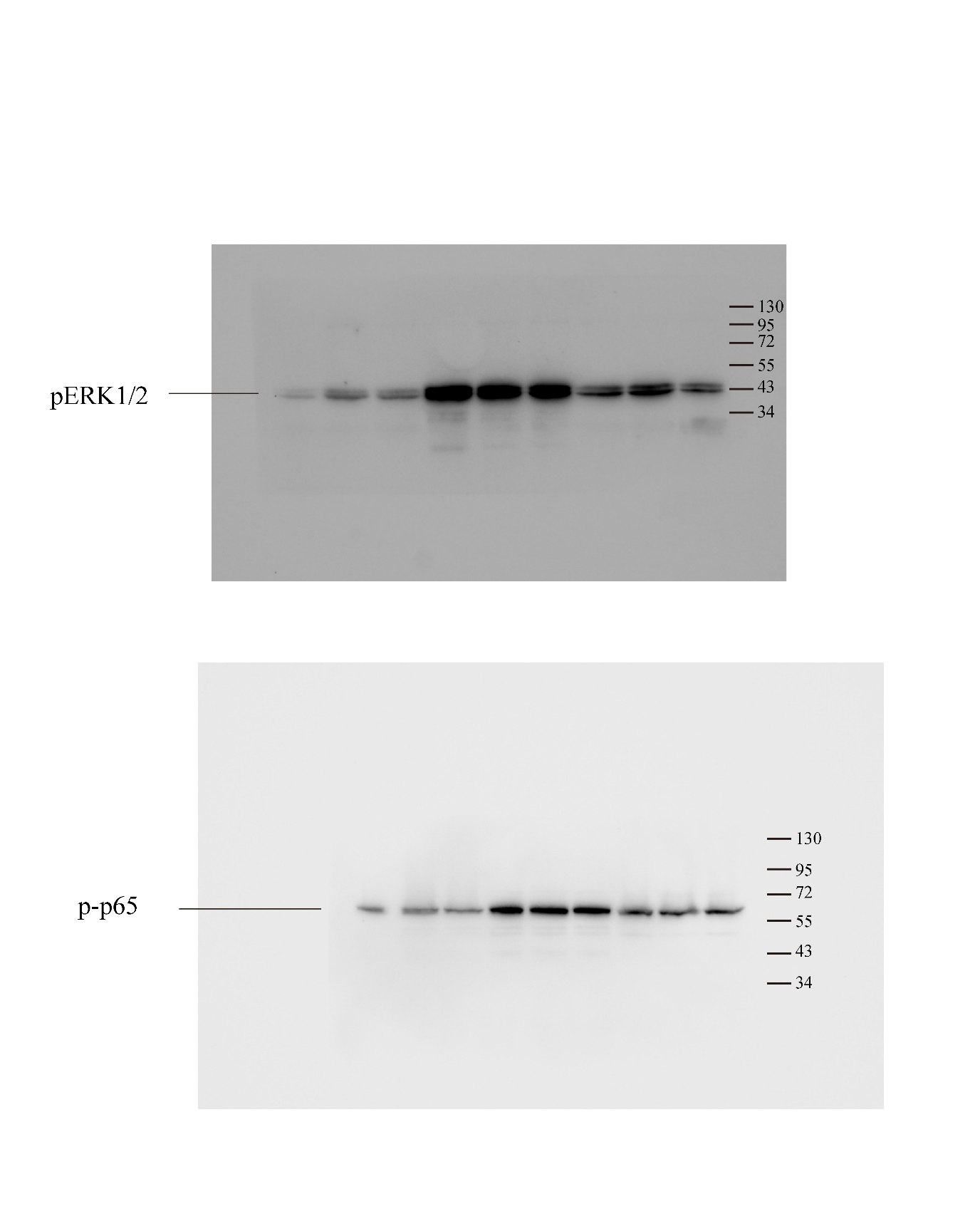


Full blots in Figure 3 (continued)


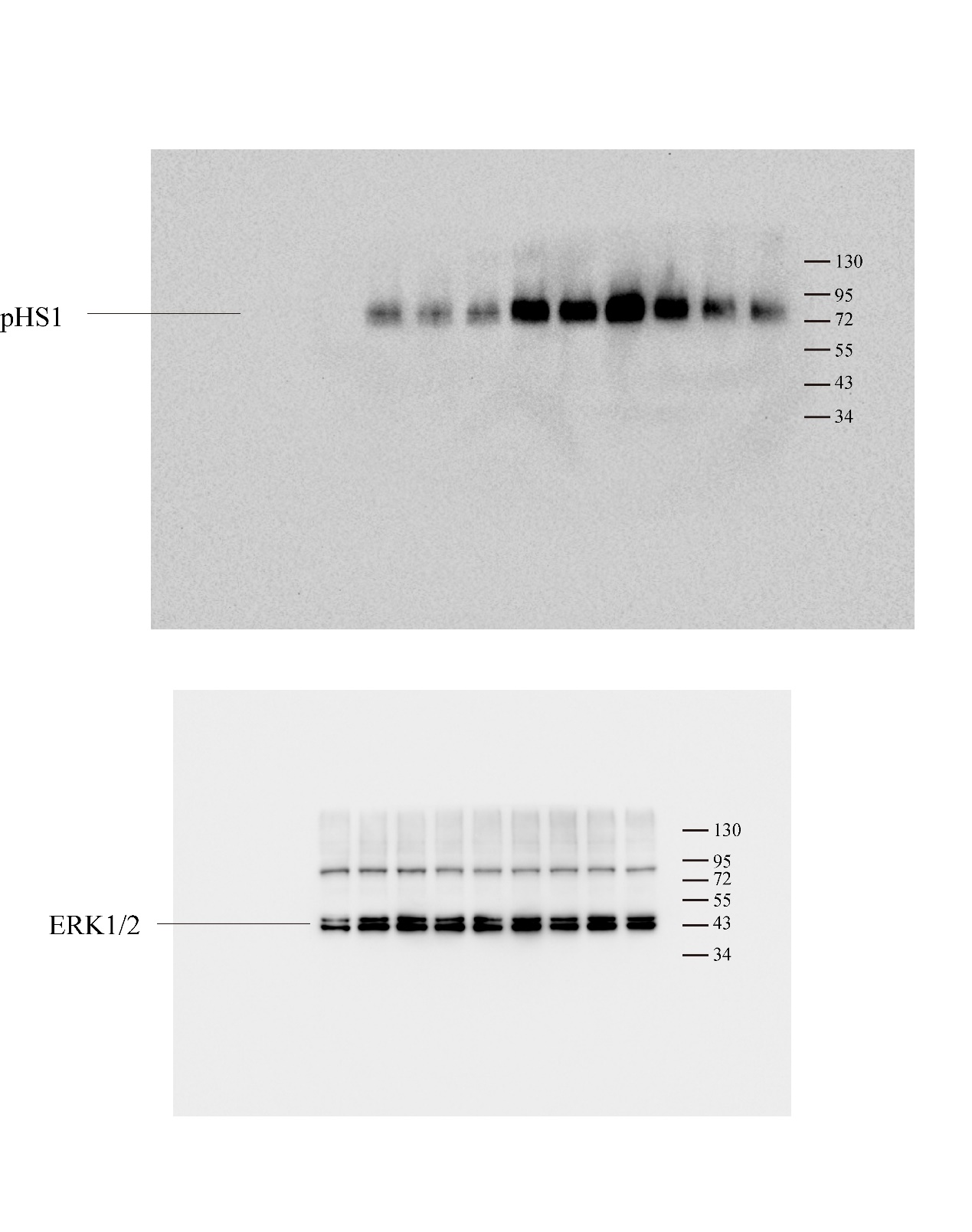


Full blots in Figure 3 (continued)


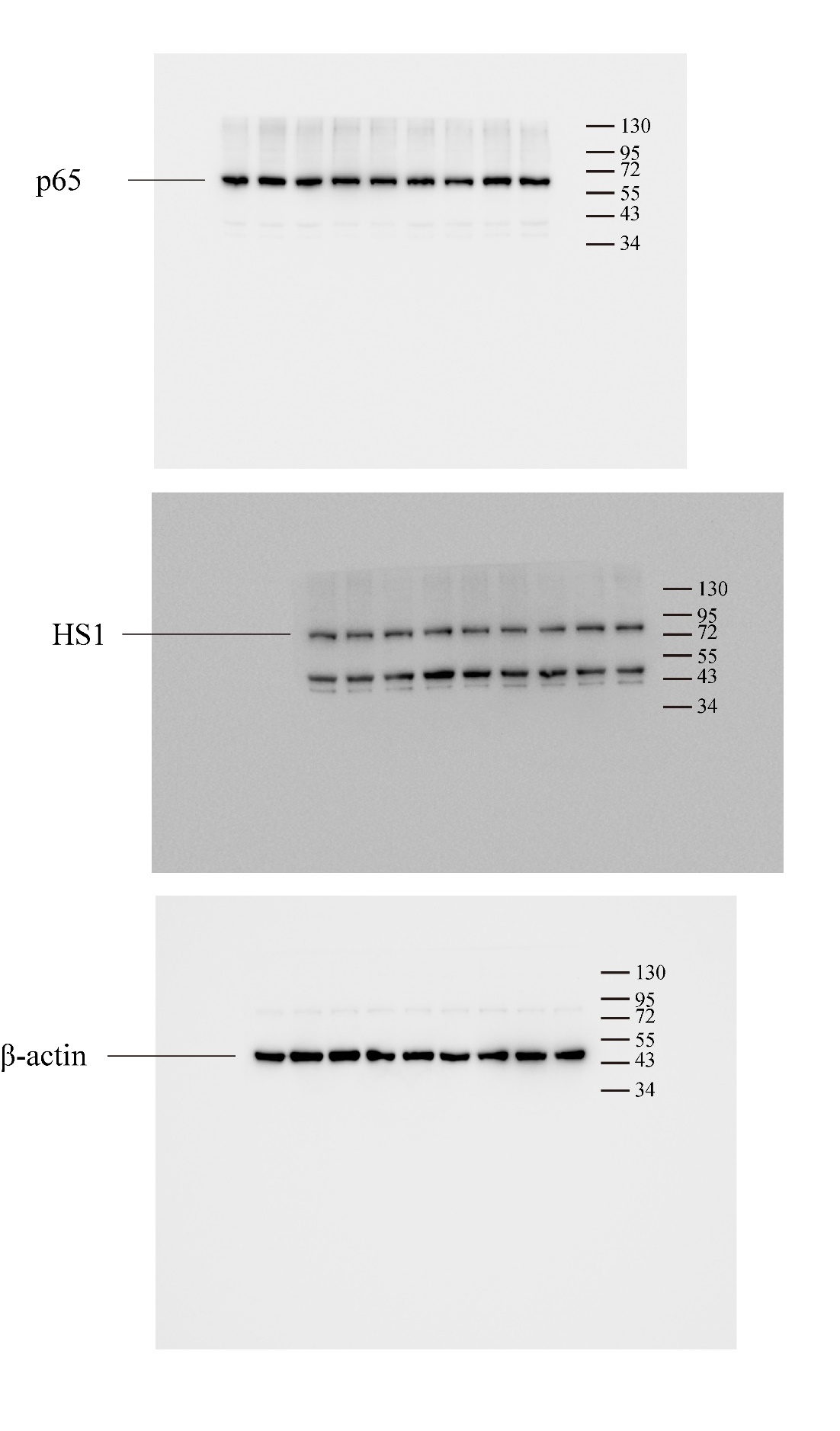


Full blots in Figure 5


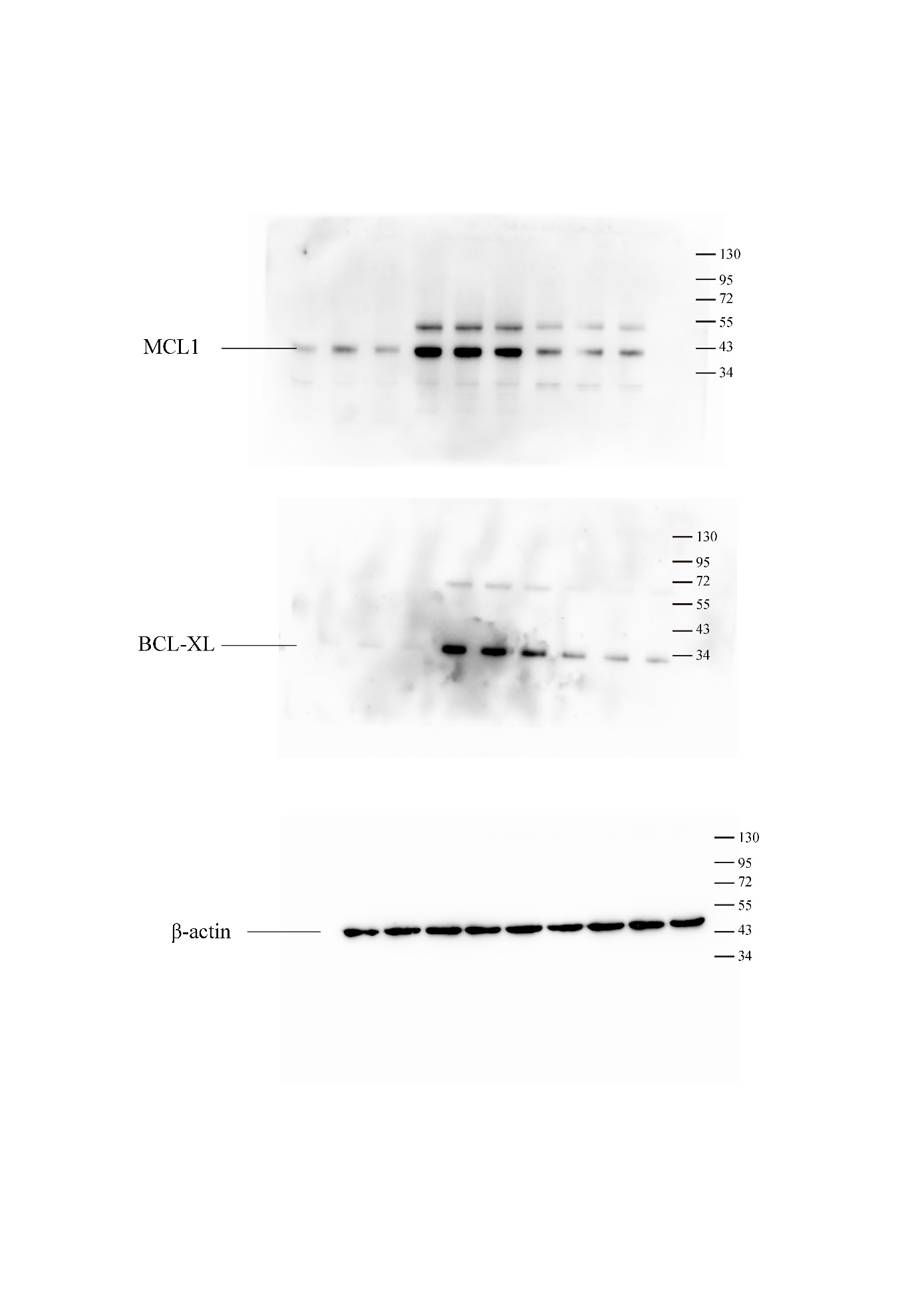


Full blots in Figure 6


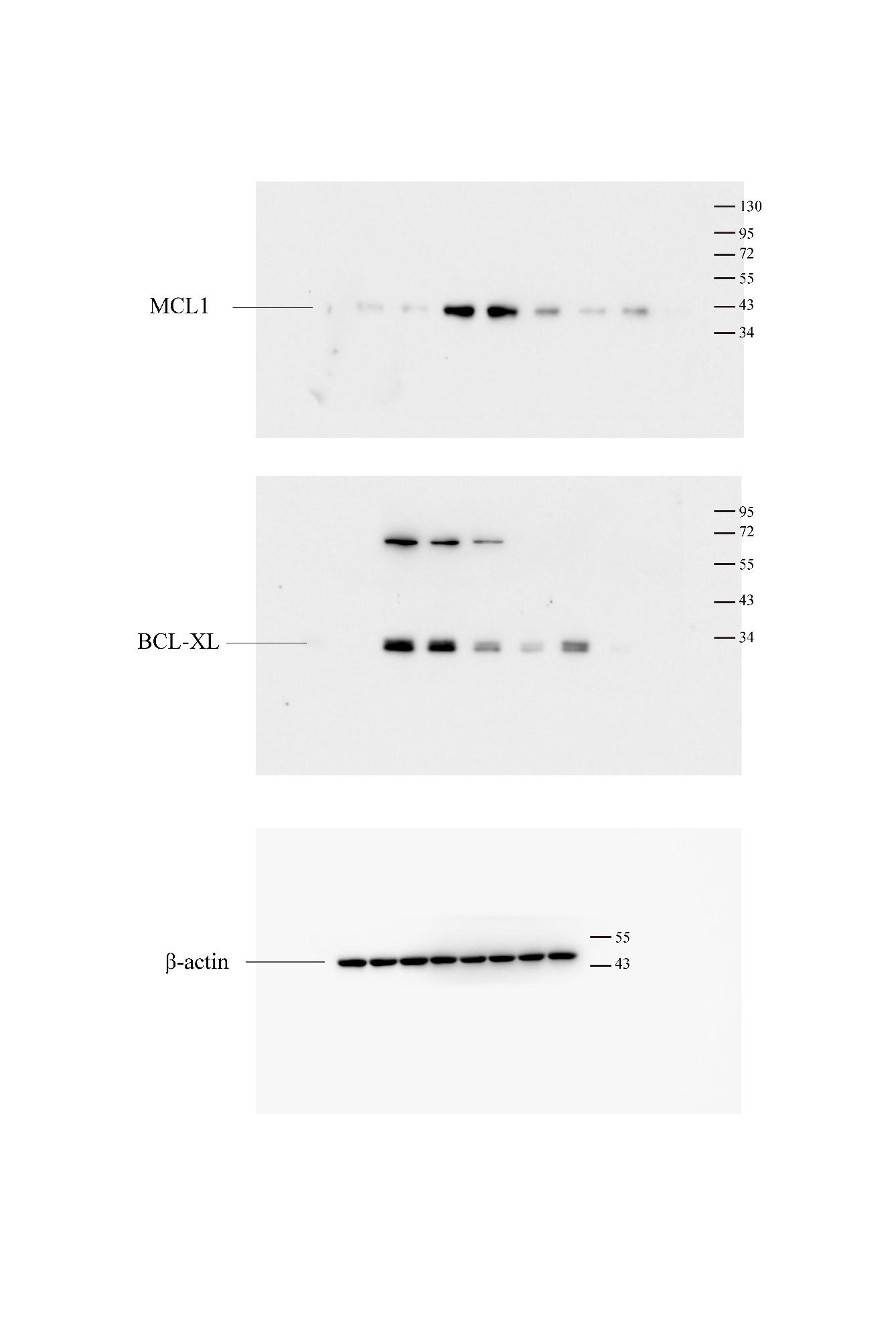

Supplement: Supplementary file 1 — Supplemental Material [file 41420_2022_884_MOESM1_ESM.docx]
